# Neoadjuvant therapy–associated malignant phenotype score predicts prognosis and highlights the roles of MIF signaling and DUXAP8 in ESCC

**DOI:** 10.3389/fimmu.2025.1683349

**Published:** 2026-01-14

**Authors:** Wenchao Xia, Tan Lin, Mengnan Shi, Qiuqiao Mu, Han Zhang, Yijun Xu

**Affiliations:** 1 Tianjin Chest Hospital and Tianjin University, Tianjin, China; 2 Qingdao Hospital, University of Health and Rehabilitation Sciences (Qingdao Municipal Hospital), Qingdao, China; 3 Department of Gastroenterology, Jining First People’s Hospital, Qingdao, China

**Keywords:** ESCC, scRNA-seq, MIF, TME, prognostic signature

## Abstract

**Background:**

Esophageal squamous cell carcinoma (ESCC) remains a major malignancy globally, and long-term survival outcomes remain poor despite advances in multimodal treatment strategies. Neoadjuvant therapy (NAT) has become the standard of care for resectable ESCC; however, substantial interpatient heterogeneity in treatment response persists. Defining malignant cell–intrinsic molecular determinants linked to NAT response is essential for improving prognostic stratification and informing individualized therapeutic decision-making.

**Methods:**

To investigate transcriptional alterations in malignant epithelial cells, we analyzed scRNA-seq datasets obtained from ESCC patients both before and after NAT. Overlapping molecular features were identified by integrating differentially expressed genes from pre- and post-treatment malignant cells with bulk RNA-seq data from TCGA and GEO cohorts. Functional enrichment analyses, pseudotime trajectory reconstruction, and transcription factor regulatory network assessments were subsequently performed. Candidate prognostic genes were initially screened through univariate Cox analysis, followed by the development of the NTAMPS model using LASSO-Cox regression. Its prognostic performance was validated in the TCGA-ESCA and GSE53624 cohorts, and clinical applicability was further examined using a nomogram, calibration curves, and decision curve analysis. Immune-related associations, immunotherapy response prediction (IMvigor210 and GSE78220), and drug sensitivity profiling were also conducted. DUXAP8, which carried the largest coefficient in the NTAMPS, was selected for further validation through both *in vivo* and *in vitro* assays, including qRT-PCR and evaluations of cell proliferation, migration, invasion, and colony formation.

**Results:**

Malignant epithelial cells exhibited pronounced transcriptional remodeling after NAT, characterized by enrichment of pathways related to the cell cycle, DNA replication, and epithelial–mesenchymal transition. Cell–cell communication analysis revealed substantial reorganization of the MIF signaling pathway, including increased interactions of MIF–ACKR3 and MIF–(CD74+CXCR4/CD44), with fibroblasts acting as major signal senders and macrophages serving as primary receivers. Twenty-one prognosis-related genes were identified, and a ten-gene NTAMPS demonstrated strong prognostic performance. Both NTAMPS and clinical stage emerged as independent prognostic factors and were integrated into a nomogram with favorable calibration and decision curve characteristics. A high NTAMPS was associated with an immunosuppressive microenvironment, reduced predicted response to immunotherapy, and distinct drug sensitivity patterns. DUXAP8, the top positive risk gene within NTAMPS, was highly expressed in ESCC tissues and cell lines, and its silencing suppressed cell proliferation, migration, invasion, and clonogenic potential.

**Conclusion:**

This study establishes NTAMPS as a novel malignant cell–derived prognostic signature for ESCC by integrating single-cell and bulk transcriptomic data. NTAMPS enables effective prognostic stratification, predicts potential immunotherapy benefit, and highlights therapeutic vulnerabilities. DUXAP8 was further identified as a candidate molecular driver that may improve ESCC management in the context of neoadjuvant therapy.

## 1 Introduction

ESCC represents a predominant histological form of esophageal cancer, comprising more than 80% of cases globally (1). In China and other East Asian countries, ESCC is a major contributor to the global burden of cancer-related illness and death (2). Owing to the lack of specific early symptoms, approximately 30% of patients present with locally advanced disease at their initial diagnosis (3, 4), typically defined as T2–T4a tumors with or without regional lymph-node involvement but without distant metastasis (M0). Patients at this stage often harbor a higher tumor burden and an increased risk of occult micrometastasis, while long-term survival after surgery alone remains poor (5). Even in the era of multimodal therapy, fewer than one-third of individuals with ESCC survive beyond five years. (6). Therefore, for patients in this critical “window period,” preoperative treatment aimed at reducing tumor burden, eliminating potential micrometastasis, and improving the likelihood of R0 resection represents an essential strategy, making them the subgroup most likely to benefit from neoadjuvant therapy.

For resectable, locally advanced ESCC, NAT is widely adopted as the standard therapeutic approach (7, 8). Both neoadjuvant chemoradiotherapy (nCRT) and neoadjuvant chemoimmunotherapy (nICT) have been demonstrated in multiple clinical trials to increase the pathological complete response (pCR) rate and prolong survival (9, 10). However, clinical practice has revealed marked interpatient heterogeneity in NAT response: some patients achieve significant tumor shrinkage and durable benefit, whereas others experience early recurrence or disease progression (5, 11). This variability suggests that conventional clinical and imaging-based staging indicators are insufficient for accurate prediction. Commonly used parameters—such as cT stage, SUVmax, and tumor length or volume—exhibit generally limited predictive performance. For example, a radiomics-based ESCC study (12) reported that tumor volume yielded an AUC of only 0.61 (95% CI: 0.46–0.76) for predicting pCR, and clinical factors including cT stage showed no significant association with treatment response. These findings indicate that traditional imaging evaluation provides suboptimal discrimination of NAT efficacy and long-term outcomes, underscoring the need for novel molecular biomarkers and risk-stratification tools.

In recent years, scRNA-seq is increasingly employed to explore tumor-intrinsic heterogeneity and the tumor microenvironment (TME) at an unparalleled level of detail (13). scRNA-seq can reveal fine-scale transcriptomic variations at the resolution of individual cells, a capability not achievable with bulk RNA sequencing, thus capturing the dynamic changes in tumor cell populations across distinct biological states, treatment responses, and evolutionary trajectories (14, 15). In ESCC research, single-cell strategies are used to characterize the immune microenvironment and delineate distinct cellular subsets implicated in immune evasion, therapy resistance, and metastasis (16, 17). Particularly in the context of NAT, scRNA-seq facilitates the tracking of transcriptional reprogramming in malignant cells and the identification of critical pathways and molecules that drive resistance and recurrence (18).

Against this background, we proposed and developed the Neoadjuvant Therapy–Associated Malignant Phenotype Score (NTAMPS), which integrates scRNA-seq and bulk transcriptomic data to quantify the molecular features of malignant cells under NAT and translate them into a clinically applicable tool for survival prediction, immunotherapy benefit assessment, and drug sensitivity estimation. We further validated the robustness of NTAMPS across multiple cohorts and analyzed its associations with TME composition, immune functional states, and genomic instability.

Moreover, risk coefficient analysis revealed that DUXAP8 exhibited the highest risk coefficient (0.26) in the NTAMPS model, suggesting its potential role in ESCC malignant progression. Accordingly, *in vitro* assays were performed to investigate how DUXAP8 influences cellular proliferation, migratory capacity, and invasive potential. This integrative strategy not only provides mechanistic insights into the heterogeneous NAT response but also offers potential targets for clinical risk assessment and personalized treatment in ESCC.

## 2 Method

### 2.1 Source and accessibility of data

In this study, we obtained the single-cell RNA sequencing (scRNA-seq) dataset PRJCA016745 (released October 25, 2024) from the Genome Sequence Archive (GSA) hosted by the National Genomics Data Center (NGDC; https://ngdc.cncb.ac.cn/bioproject/browse/PRJCA016745). This dataset contains single-cell profiles from 22 ESCC patients who underwent neoadjuvant treatment with combined chemotherapy and immunotherapy prior to surgical resection. Bulk RNA-seq profiles with corresponding clinical annotations for ESCC were retrieved via the TCGAbiolinks package in R from the TCGA-ESCA dataset (https://portal.gdc.cancer.gov/) (19). For external validation, the GSE53624 dataset was obtained from the GEO database (https://www.ncbi.nlm.nih.gov/geo/).

### 2.2 Workflow for single-cell data analysis

Processing of single-cell transcriptomic data was performed with Seurat (v4.4.0) (20). The 10x Genomics-format matrix files (barcodes/genes/matrix) for each sample were imported and merged into a unified Seurat object. Metrics including transcript counts (nCount_RNA), detected gene numbers (nFeature_RNA), and the proportions of mitochondrial (pMT) and hemoglobin (pHB) genes were computed. Cells passing quality control thresholds (500 ≤ nFeature_RNA ≤ 10,000; 1,000 ≤ nCount_RNA ≤ 100,000; pMT < 40%; pHB < 5%) were retained for downstream analyses. Normalization was performed using the NormalizeData function, and the top 3,000 variable genes were identified with the VST approach. Cell cycle scoring for S and G2M phases was performed using predefined gene sets in Seurat, and cell cycle scores were also regressed during scaling. PCA was applied for dimensionality reduction, and batch effects were adjusted with Harmony, grouping cells by sample ID. The first 20 Harmony dimensions were used to construct the nearest-neighbor graph, perform clustering at a resolution of 1.0, and generate UMAP embeddings for visualization. Cluster-specific marker genes were detected with FindAllMarkers, and cell identities were assigned based on published references and a curated annotation table. For visualization, each cell type was randomly downsampled to a maximum of 8,374 cells, and the top 10 marker genes were selected to generate a heatmap.

### 2.3 Evaluation of intercellular signaling networks

Cell–cell communication networks were inferred using CellChat (21). Samples were divided into pre-treatment and post-treatment groups based on clinical information, and major cell types were used as identity labels for analysis. Known human ligand–receptor interaction databases were referenced to preprocess the expression data, including the identification of overexpressed ligands and receptors, and mapping to protein–protein interaction networks to enhance prediction accuracy. Communication probabilities were calculated for each ligand–receptor pair, followed by statistical significance testing and removal of clusters with insufficient cell numbers. At the pathway level, communication strength and the number of interactions were aggregated, and centrality measures were computed to determine the roles of each cell type in sending and receiving signals. Finally, the two groups were compared to assess changes in communication patterns, and key signaling pathways and ligand–receptor pairs showing significant differences under different conditions were identified.

### 2.4 Evaluation of genomic copy number alterations in epithelial cells

We employed inferCNV (v1.22.0) (22) to evaluate epithelial cell malignancy, using raw count matrices derived from the Seurat object. Cell types were designated as malignant or normal according to tissue origin, taking normal epithelial cells as the reference. Genomic ordering was based on the human gene position reference file (hg19). The analysis was run with the following parameter settings: cutoff = 0.1, cluster_by_groups = TRUE, denoise = TRUE, and HMM disabled. Copy number variation (CNV) scores were computed for each cell by rescaling expression values and calculating mean squared deviations. Correlation coefficients with the top 5% of high-CNV malignant cells were used, together with CNV score thresholds, to classify cells as “Cancer” or “Normal.” The classification results were integrated into the Seurat object metadata for downstream analyses.

### 2.5 Pseudotime trajectory analysis using monocle 2

For malignant epithelial cells, pseudotime trajectories were constructed using Monocle 2 (v2.32.0) (23) based on UMI count matrices. Size factors and dispersion parameters were first estimated, and genes were filtered with a minimum expression threshold of 0.1 and expression present in no fewer than 10 cells. Differential expression analysis was performed with cell type as the grouping variable, and the top 2,000 significant genes were selected as ordering genes. Dimensionality reduction employed the DDRTree algorithm configured with three centers, followed by cell ordering to derive pseudotime and state information. To investigate transcriptional changes at branch points, branch-dependent gene analysis was performed at the first branch, and functional interpretation was conducted through Gene Ontology biological process enrichment. Trajectory and heatmap visualizations were used to illustrate overall trends without inclusion in statistical inference.

### 2.6 Transcription factor analysis

The transcriptional regulatory network of malignant epithelial cells was characterized using the SCENIC (Single-Cell rEgulatory Network Inference and Clustering) workflow (24). First, GENIE3 was applied to infer putative transcription factor–target gene interactions based on gene co-expression patterns. Next, RcisTarget was employed to identify significantly enriched DNA-binding motifs among the predicted target genes, thereby refining the regulon definitions. The activity of each regulon was then assessed at single-cell resolution via AUCell, which determines AUC values from the ranked expression of regulon-specific target genes per cell.

Regulon activity matrices were integrated with the cell state annotations to identify transcription factors preferentially activated in specific functional states of malignant epithelial cells. Visualization, including heatmaps and bubble plots, was used to highlight key transcription factors and their associated regulatory programs across cell states.

### 2.7 hdWGCNA-based detection of co-expressed gene modules

Weighted gene co-expression networks were constructed for malignant epithelial cells using the hdWGCNA/WGCNA framework (25). Genes detected in no fewer than 5% of cells were kept for downstream analysis. Cells were aggregated into metacells (~25 similar cells per metacell) according to cell subtypes and sample origins, followed by normalization of the metacell expression matrix. Soft-threshold power selection followed the scale-free topology principle (signed network, power = 14), and modules were, and modules were identified under a merge cut height of 0.4. Module eigengenes (hMEs) were calculated across samples, module connectivity was assessed, and hub genes were defined according to kME values. Module expression scores were computed at the single-cell level (UCell) for the top 25 hub genes per module. Visualizations such as soft-threshold plots, dendrograms, module correlations, hub-gene networks, and UMAP distributions were generated to illustrate the overall structure of the networks, without being used for statistical inference.

### 2.8 Design and optimization of the predictive model

The overlap of differentially expressed genes identified from the TCGA cohort (DEGs_TCGA), the GEO cohort (DEGs_GEO), and single-cell datasets comparing pre- and post-neoadjuvant therapy was determined to generate a candidate gene list. Univariate Cox regression was applied to these overlapping genes to identify those significantly linked to overall survival. Subsequently, the least absolute shrinkage and selection operator (LASSO) method was used for variable selection and to address multicollinearity, followed by stepwise Cox regression to construct the final multi-gene prognostic signature. The risk score for each patient was calculated using the following formula:


$$Risk\;Score=\beta_{1}\times Exp_{1}+\beta_{2}\times Exp_{2}+\ldots +\beta_{n}\times Exp_{n}$$

where β represents the regression coefficient from the multivariate Cox regression, Exp denotes the expression level of the corresponding gene, and n is the number of genes included in the model.

Patients were stratified into high- and low-risk groups according to the median value of the calculated risk score. Kaplan–Meier survival curves were generated, and differences in overall survival between the two groups were assessed using the log-rank test. To evaluate the predictive performance of the model, time-dependent receiver operating characteristic (ROC) curves (26) were plotted for 1-, 3-, and 5-year survival, and the area under the curve (AUC) was calculated to quantify discriminatory ability.

### 2.9 Establishment of a multi-parameter prognostic nomogram

Univariate and multivariate Cox regression analyses were performed using the risk score, sex, and pathological stage to identify variables independently associated with prognosis. These factors were integrated into a nomogram for estimating overall survival at 1, 3, and 5 years. The agreement between predicted and observed survival was examined using calibration curves, with internal validation conducted through bootstrap resampling. Decision curve analysis (DCA) was applied to evaluate net clinical benefit across varying decision thresholds. The nomogram’s predictive accuracy was also benchmarked against models constructed from single parameters to highlight its incremental value.

### 2.10 Assessment of immunotherapy outcomes and drug sensitivity

To explore the clinical relevance of the prognostic signature in the immunotherapy setting, the model was applied to two publicly available cohorts of patients who had received immune checkpoint inhibitors (IMvigor210 and GSE78220). Prognostic associations were examined, and differences in risk score distributions were compared between clinical response categories. Single-sample gene set enrichment analysis (ssGSEA) was then employed to estimate enrichment levels for immune_cycle and predicted immunotherapy-related pathways (27), followed by correlation analysis with the risk score.

For pharmacogenomic evaluation, the GDSC2 (28) resource was utilized. Expression matrices underwent preprocessing to merge duplicated genes, filter out low-abundance transcripts, and remove non-tumor samples. The calcPhenotype function from the oncoPredict package (29), trained on GDSC2 drug response profiles, was used to estimate the half-maximal inhibitory concentration (IC50) of a range of anticancer agents for each patient, enabling comparison of drug sensitivity patterns across risk subgroups.

### 2.11 Measurement of gene expression by RT-qPCR

ESCC tumor tissues were obtained from patients undergoing surgical resection at Tianjin Chest Hospital, following approval by the institutional Ethics Committee. Freshly excised specimens were snap-frozen in liquid nitrogen and stored until RNA extraction. Total RNA was extracted using TRIzol reagent (Invitrogen, USA) per the manufacturer’s guidelines, and its purity and concentration were determined. Reverse transcription to cDNA was carried out with a Takara kit (Japan). Quantitative real-time PCR was performed on an ABI 7500 platform (Applied Biosystems, USA) under the kit-recommended cycling conditions. GAPDH was used as an internal reference, and relative gene expression was quantified using the 2^-ΔΔCt method. The primer sequences used for amplification are provided in **Supplementary Table 1**.

### 2.12 siRNA transfection procedures for ESCC cell lines

KYSE-150, KYSE-410, and T_HEECS cell lines were sourced from the Cell Bank of the Chinese Academy of Sciences (Shanghai, China), verified by short tandem repeat (STR) analysis, and confirmed free of mycoplasma. Cells were cultured in basal medium containing 10% fetal bovine serum and 1% penicillin–streptomycin, maintained at 37 °C in a 5% CO_2_ humidified incubator. Upon reaching 30%–60% confluence, transfection with target-specific or control siRNAs was carried out using a liposome-based approach. Six to eight hours later, the medium was replaced with fresh complete medium. Forty-eight hours post-transfection, cells were harvested, and total RNA was extracted for RT-qPCR to determine transfection efficiency by measuring target gene expression. siRNA sequences are provided in **Supplementary Table 1**.

### 2.13 Assessment of cell migration and invasion *in vitro*

Cell motility and invasiveness were assessed using 24-well Transwell inserts with 8-μm pores. For migration assays, cells suspended in serum-free medium were added to the upper compartment, while the lower chamber contained 10% FBS as a chemoattractant. For invasion assays, membranes were coated with Matrigel prior to seeding. Following incubation (16–24 h for migration; 24–48 h for invasion), cells remaining on the upper surface were removed, and those on the lower surface were fixed, crystal violet–stained, and enumerated microscopically.

### 2.14 Cell proliferation assay using CCK-8

Control and siRNA-transfected cells were plated into 96-well dishes at a density of 2–5 × 10³ cells per well. Each day from Day 1 to Day 6, 10 μL of CCK-8 solution was applied and incubated for 1–2 h at 37 °C. Optical density at 450 nm was recorded with a microplate reader, and proliferation curves were generated based on triplicate wells from three separate experiments.

### 2.15 Colony formation assay

Both control and siRNA-transfected cells were detached using trypsin, counted, and plated in six-well dishes at a density of 500–1,000 cells per well. Cultures were maintained at 37 °C in 5% CO_2_ for 10–14 days, replacing the medium periodically. Formed colonies were rinsed with PBS, fixed in methanol, stained with 0.1% crystal violet, washed, dried, and enumerated when reaching ≥1 mm in diameter or comprising ≥50 cells.

### 2.16 Statistical methodology

All statistical analyses were conducted using R software (version 4.3.2). Continuous variables were expressed as mean ± standard deviation (mean ± SD). Comparisons between two groups were performed using the two-tailed independent Student’s t-test or the Wilcoxon rank-sum test, while multiple group comparisons were assessed using one-way ANOVA or the Kruskal–Wallis test. Survival analyses were conducted with the Kaplan–Meier method and log-rank tests, and univariate and multivariate Cox proportional hazards regression analyses were applied to identify prognostic factors. Depending on the data distribution, correlations were assessed using Spearman’s or Pearson’s approaches. All statistical tests were two-tailed, with significance defined as P < 0.05.

## 3 Results

### 3.1 Comprehensive single-cell landscape and annotation of ESCC samples

Prior to downstream analysis, raw single-cell sequencing data underwent stringent quality control. Following quality control filtering, noticeable enhancements were observed in sequencing depth (nCount), gene detection counts (nFeature), and the percentage of mitochondrial transcripts (pMT) relative to pre-QC values (**Supplementary Figures S1A, B**), ensuring data reliability and analytical robustness.

**Figure 1:**
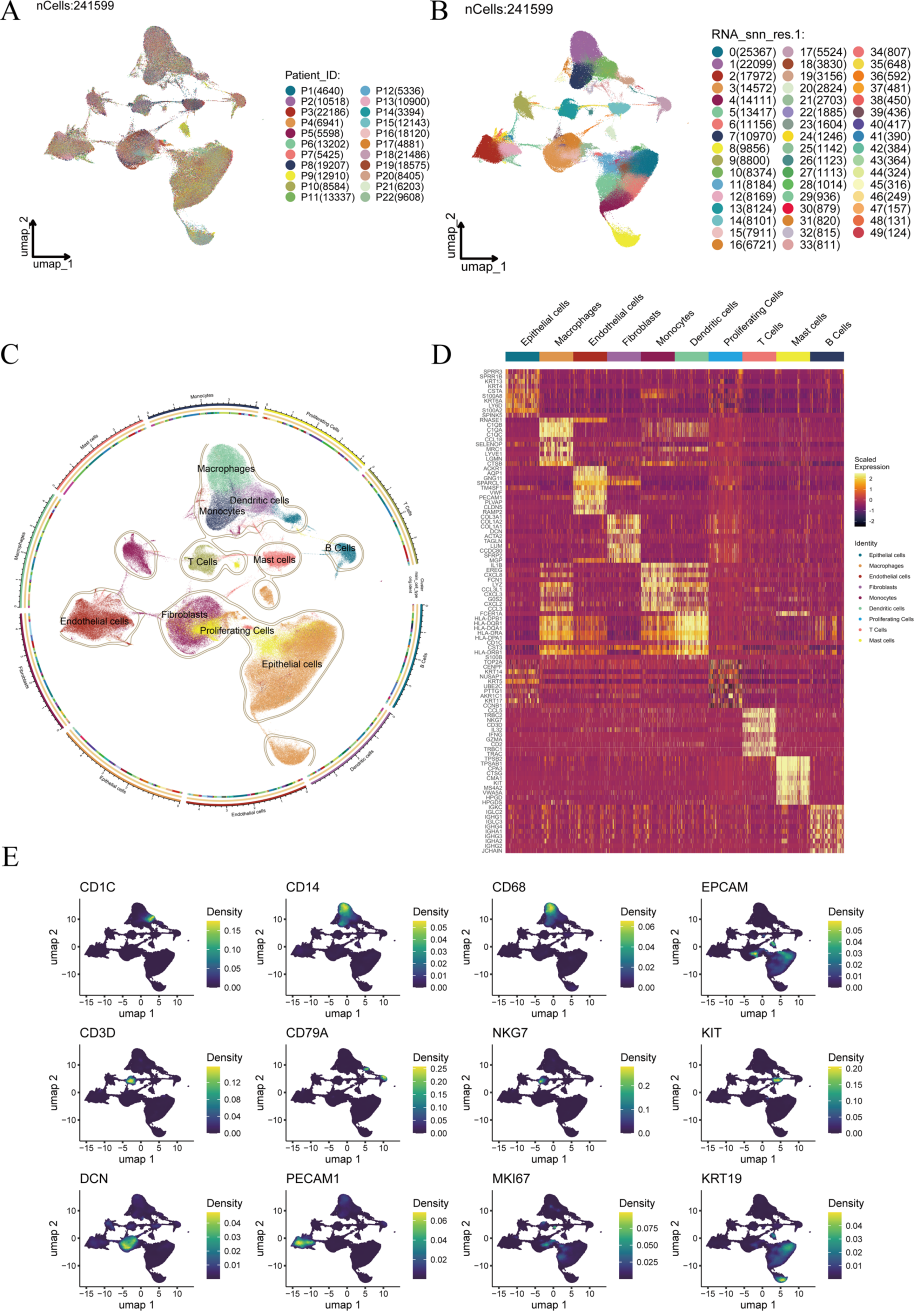
Single-cell transcriptomic landscape and cell-type annotation of ESCC samples. **(A)** UMAP visualization of cells colored by patient origin. **(B)** UMAP plot showing clustering results. **(C)** Annotated cell types projected onto the UMAP embedding. **(D)** Heatmap of the top 10 highly expressed genes in each cell type. **(E)** Distribution of representative marker genes across annotated cell types.

Integration and dimensionality reduction of all samples yielded a total of 241,599 high-quality cells. Visualization in UMAP space revealed a well-mixed distribution across patient samples, with no obvious batch effects (**Figure 1A**). An initial unsupervised clustering step partitioned these cells into 50 transcriptionally distinct clusters (**Figure 1B**). Subsequent annotation, guided by differentially expressed genes and canonical markers, consolidated these clusters into major functional cell types, including T cells, B cells, NK cells, monocytes, macrophages, dendritic cells, fibroblasts, endothelial cells, epithelial cells, and proliferating cells (**Figure 1C**). Marker gene expression patterns for each cluster are displayed in a heatmap (**Supplementary Figure S1C**), validating the accuracy of the cell-type assignments. Heatmap visualization (**Figure 1D**) revealed unique expression profiles for the ten most abundant genes in each cell type, showing strong enrichment within their respective populations. Representative marker genes were further visualized in UMAP space (**Figure 1E**), where their spatial expression patterns closely aligned with the annotated cell identities, providing a robust foundation for subsequent functional analyses.

### 3.2 Examination of intercellular communication and MIF pathway dynamics before and after treatment

**Figure 2:**
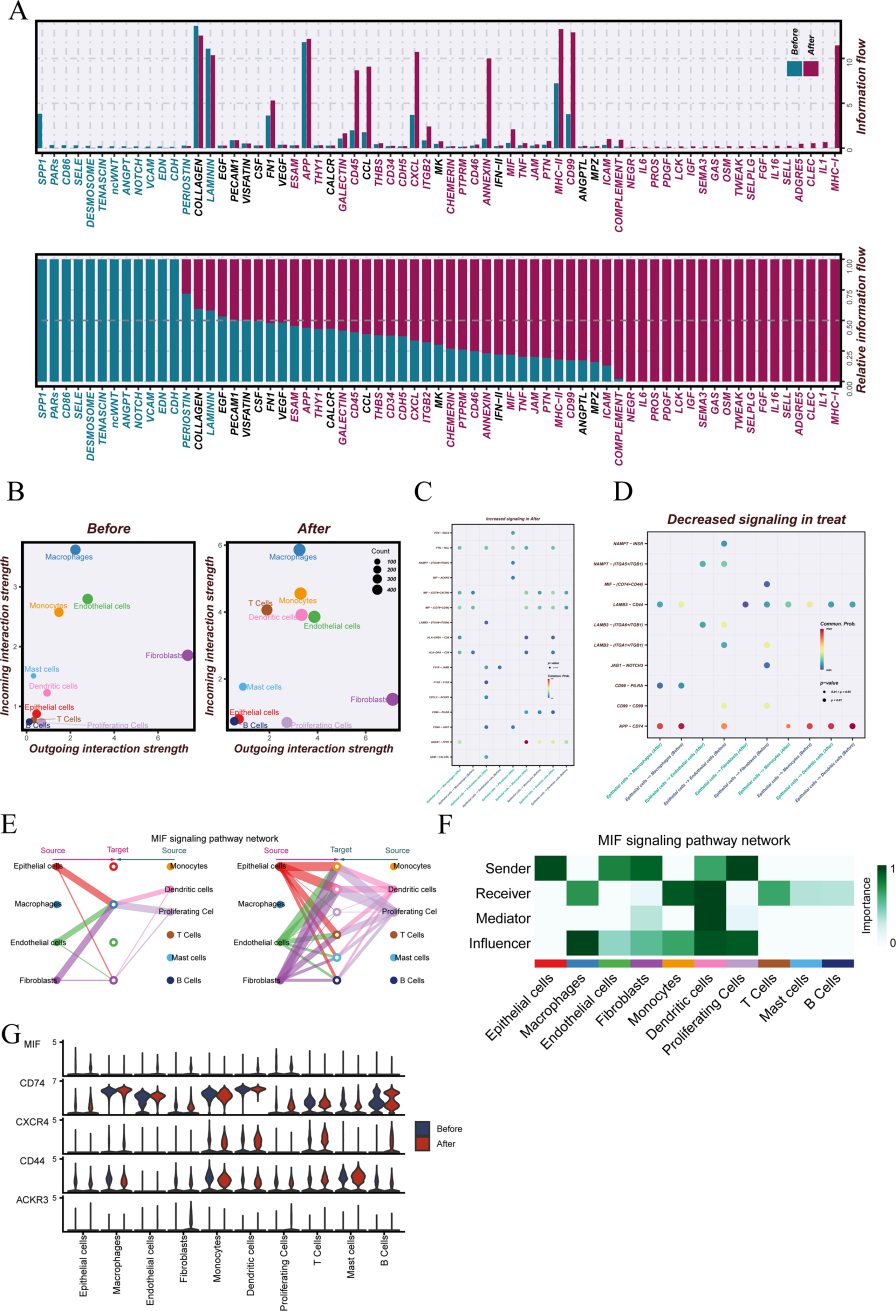
Analysis of cell–cell communication networks and MIF signaling pathways in pre- and post-treatment samples. **(A)** Comparative landscape of cell–cell communication networks before and after treatment, revealing global changes in intercellular signaling activity. **(B)** Quantitative assessment of the relative contribution of each cell type to overall signaling activity under pre- and post-treatment conditions. **(C)** Signaling pathways with increased activity in the post-treatment group, with epithelial cells designated as the primary signal senders. **(D)** Signaling pathways with decreased activity after treatment when epithelial cells act as the main signal source. **(E)** Summary of the main biological functions of the macrophage migration inhibitory factor (MIF)-mediated signaling cascade. **(F)** Schematic representation of MIF-driven signaling interactions, illustrating its connections with key target cell types. **(G)** Patterns of MIF-associated gene expression in various cell populations in pre- and post-treatment samples.

In-depth examination of intercellular communication revealed substantial alterations in signaling pathway activities between pre- and post-treatment samples (**Figure 2A**). The most pronounced changes were observed in SPP1, PARs, ESAM, MIF, MHC−II, and MHC−I signaling, participating in processes including cellular adhesion, immune activation, and inflammatory regulation. These findings suggest that treatment reshapes the tumor microenvironment through multiple intercellular signaling routes.

The overall signaling strength for individual cell types is presented in **Figure 2B**. Macrophages consistently emerged as the strongest signal receivers in both groups, occupying central positions in several immune- and inflammation-related pathways. Conversely, fibroblasts were the dominant signal senders before and after treatment, particularly contributing to extracellular matrix remodeling–associated signaling. This stable network structure indicates a sustained central role of macrophages and fibroblasts in microenvironmental communication.

When focusing on epithelial cells as signal senders (**Figures 2C, D**), the most prominent increases in activity post-treatment were found in MIF-related signaling pairs, including MIF − ACKR3, MIF − (CD74+CXCR4), and MIF − (CD74+CD44), suggesting a potential role in enhancing immune cell recruitment and activation. Conversely, the MIF − (CD74+CD44) pair was also detected among the pathways with reduced activity, indicating complex regulatory dynamics during therapy. Based on these observations, the MIF pathway was selected for detailed downstream analysis.

Functional enrichment of MIF signaling (**Figure 2E**) highlighted its involvement in immune cell migration, cytokine secretion, and cell survival. Pairwise interaction mapping (**Figure 2F**) demonstrated that MIF was primarily derived from epithelial and certain immune cell populations, interacting with receptors such as CD74, CXCR4, and CD44, with several key interactions strengthened following treatment. Corresponding gene expression profiles (**Figure 2G**) confirmed the upregulation of these ligands and receptors post-treatment, supporting their potential role in immune modulation and tumor microenvironment remodeling.

### 3.3 Identification and functional characterization of tumor-associated epithelial populations

**Figure 3:**
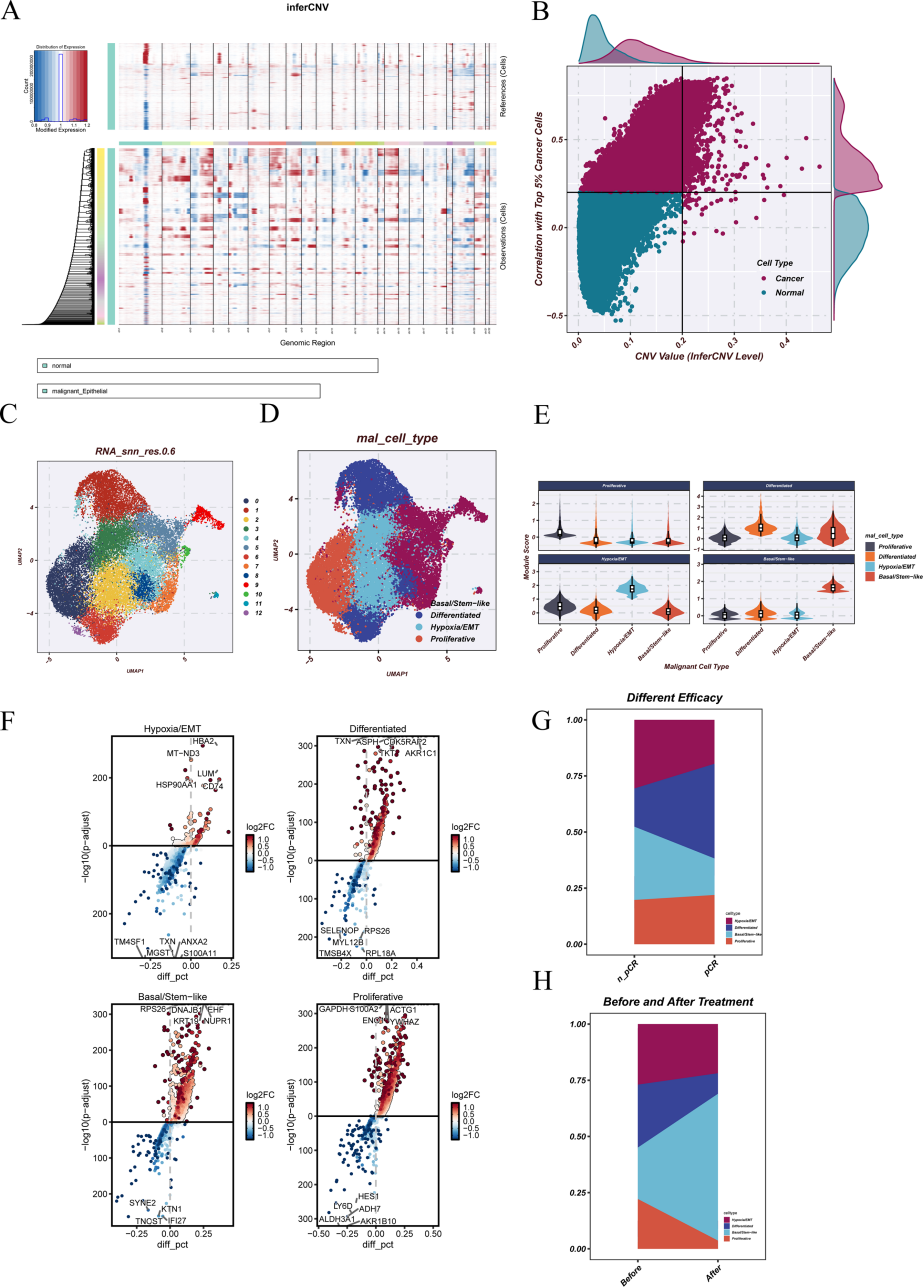
Identification and functional characterization of malignant epithelial cells. **(A)** InferCNV analysis showing large-scale chromosomal copy number variation (CNV) profiles in epithelial cells, with red and blue indicating genomic amplifications and deletions, respectively. **(B)** Correlation between CNV levels and the proportion of high-CNV cells, used to distinguish malignant from normal epithelial cells. **(C)** Clustering of malignant epithelial cells, where transcriptional features were used to divide malignant cells into multiple subclusters. **(D)** Functional state–based cell annotation, in which malignant epithelial cell subclusters were annotated according to functional state scores calculated using the AddModuleScore method. **(E)** Functional state scores calculated by AddModuleScore, showing the distribution of scores for various biological pathways and functional states across malignant epithelial subclusters. **(F)** Differentially expressed genes (DEGs) in malignant epithelial cells, with volcano plots highlighting representative genes significantly upregulated or downregulated under different clinical contexts. **(G)** Changes in the proportion of malignant epithelial cells among patients with different therapeutic responses. **(H)** Changes in the proportion of malignant epithelial cells before and after treatment.

Using copy number variation (CNV) profiles of epithelial cells, inferCNV analysis revealed distinct large-scale chromosomal amplifications and deletions in a subset of cells, indicating the presence of potential malignant clones (**Figure 3A**). The reference profile was defined from cells ranking within the highest 5% for CNV scores, after which Pearson correlation coefficients were computed by comparing each cell’s CNA profile with this reference set, ensuring consistency in the classification proces. Cells were classified as malignant when either the CNA_value > 0.2 or the correlation coefficient r > 0.2, resulting in a clear separation between malignant and non-malignant populations in the two-dimensional scatter plot (**Figure 3B**). Unsupervised clustering of malignant epithelial cells identified 13 transcriptionally distinct subclusters (**Figure 3C**).

To characterize their functional states, we applied AddModuleScore based on predefined marker gene sets corresponding to multiple functional programs, and annotated the cells as Hypoxia/EMT, Differentiated, Basal/Stem-like, or Proliferative (**Figure 3D**). A full listing of the marker genes corresponding to each functional state is available in **Supplementary Table 2**. The scoring results demonstrated clear differences among clusters, supporting the accuracy of the annotations (**Figure 3E**). Subsequent differential expression analysis revealed representative genes for each functional state, highlighting their molecular distinctions (**Figure 3F**). When comparing groups with different clinical responses, the poor-response group was enriched for Hypoxia/EMT and Basal/Stem-like cells, whereas the good-response group contained a higher proportion of Differentiated and some Proliferative cells (**Figure 3G**). In paired pre- and post-treatment samples, the proportion of Basal/Stem-like cells markedly increased after treatment, while Hypoxia/EMT, Differentiated, and Proliferative populations all decreased (**Figure 3H**).

Collectively, these findings reveal a functional state reprogramming of malignant epithelial cells across different response groups and treatment stages, suggesting that specific functional states may play pivotal roles in drug resistance and disease progression.

### 3.4 Pseudotime trajectory and transcription factor landscape of malignant epithelial cells

**Figure 4:**
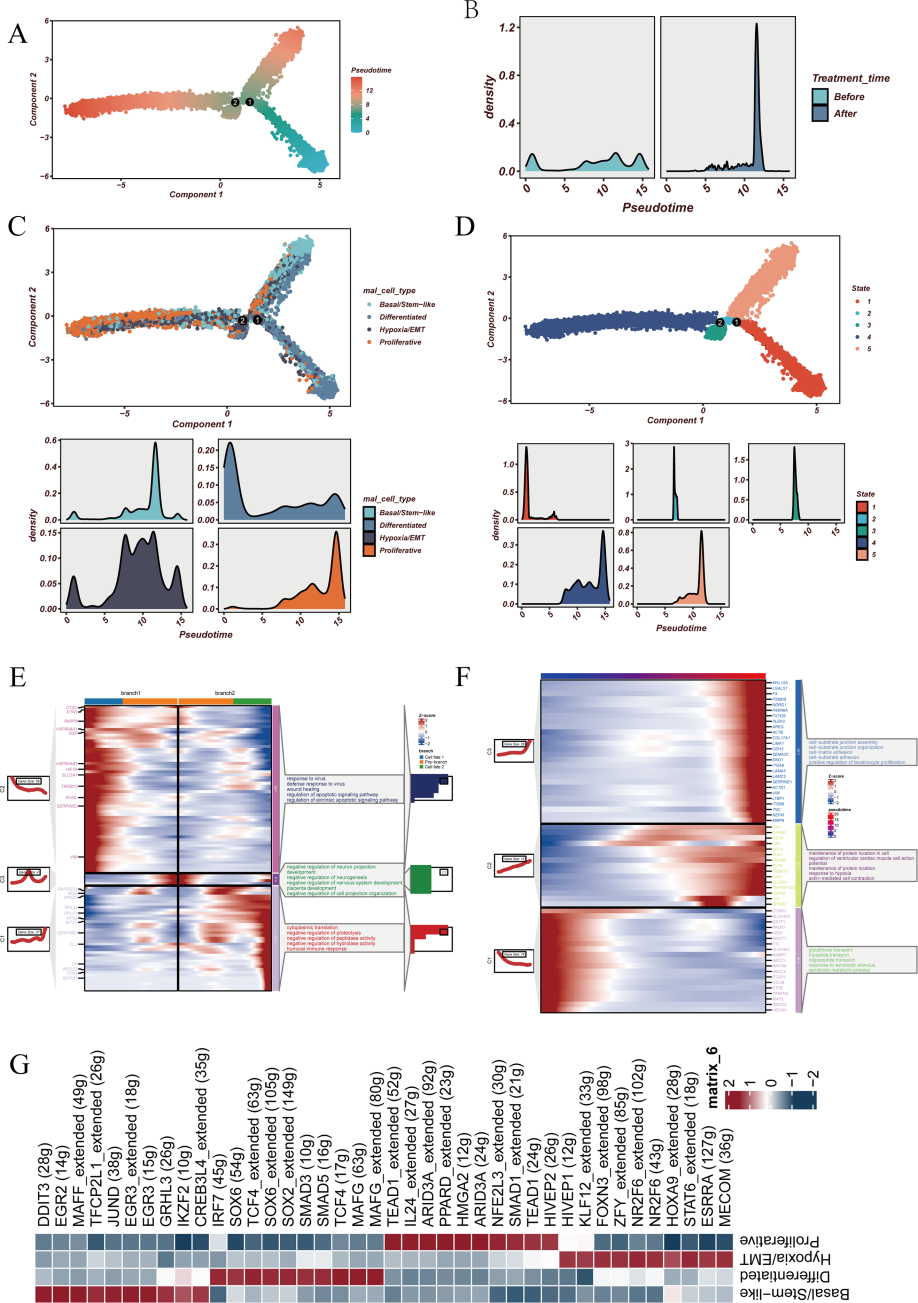
Pseudotime trajectory and transcription factor analysis. **(A)** Pseudotime differentiation trajectory of malignant epithelial cells constructed using Monocle2. The x- and y-axes represent the two principal components obtained after dimensionality reduction, with colors indicating cell positions along pseudotime, revealing a continuous progression from early to late differentiation states. **(B)** Density distribution of cells at different treatment stages (before vs. after treatment) along pseudotime, illustrating potential shifts in the temporal distribution of cells induced by treatment. **(C)** Distribution of cells with four functional states (Hypoxia/EMT, Differentiated, Basal/Stem−like, and Proliferative) along the pseudotime trajectory. The lower panels depict the density curves for each state, reflecting their dynamic changes during the differentiation process. **(D)** Pseudotime distribution of the five differentiation states (State 1–5) identified from the trajectory, along with their corresponding density plots. **(E)** At the key branching point (State 1) of the trajectory, cells were divided into two differentiation branches (Branch 1 and Branch 2). BEAM (branch-specific expression analysis) was applied to identify differentially expressed genes with unique enrichment, and subsequent GO and KEGG enrichment analyses were performed to determine the major biological processes and signaling pathways associated with each differentiation trajectory. **(F)** Heatmap illustrating changes in key gene expression across pseudotime, together with GO and KEGG enrichment analyses to clarify their potential functions and underlying regulatory mechanisms during differentiation. **(G)** Heatmap of transcription factors linked to malignant epithelial cells across four functional states, highlighting distinct activity patterns and potential regulatory roles across states.

Pseudotime and transcription factor analyses were performed to delineate the dynamic transitions of malignant epithelial cells and uncover potential regulatory mechanisms driving functional state reprogramming (**Figure 4A**). The pseudotime trajectory revealed a continuous progression from low- to high-differentiation states. Incorporating treatment status showed that pre-treatment cells were more evenly distributed along the trajectory, whereas post-treatment cells predominantly accumulated at the terminal differentiation stage (**Figure 4B**). Mapping functional states onto the trajectory (**Figure 4C**) demonstrated that Proliferative and Basal/Stem−like cells were enriched at the late differentiation stage, Hypoxia/EMT cells were primarily positioned at the early stage, and Differentiated cells occupied intermediate positions. Five distinct differentiation states were identified along the trajectory (**Figure 4D**). At state 1, two major branches emerged (**Figure 4E**). Functional enrichment indicated that one branch was associated with tissue repair and apoptotic signaling regulation, while the other was enriched for immune regulation, protein synthesis, and negative regulation of hydrolase activity. The intermediate state was primarily linked to negative regulation of neurogenesis. Dynamic gene expression analysis across the trajectory (**Figure 4F**) revealed that late-stage differentiation was characterized by activation of pathways related to cell–substrate adhesion and junction organization, whereas early-stage differentiation was enriched in processes such as oligopeptide transport and xenobiotic stimulus response.

Finally, transcription factor network analysis (**Figure 4G**) identified distinct regulatory programs across the four functional states of malignant epithelial cells, suggesting that specific transcription factors may play pivotal roles in orchestrating cell state transitions.

### 3.5 Integrative hdWGCNA reveals functional modules and hub genes in malignant epithelial cell states

To further elucidate the transcriptional co-variation patterns of malignant epithelial cells with distinct functional states and to identify key genes driving their phenotypic features, high-dimensional weighted gene co-expression network analysis (hdWGCNA) was conducted at the single-cell level. Compared with single-gene differential analysis, hdWGCNA groups functionally related genes into the same module based on expression correlations, thereby capturing underlying biological processes and signaling pathways. This network-based approach is particularly suited for exploring transcriptional regulatory modules within specific functional states and for uncovering their cell type–specific distribution and potential core regulators.

**Figure 5:**
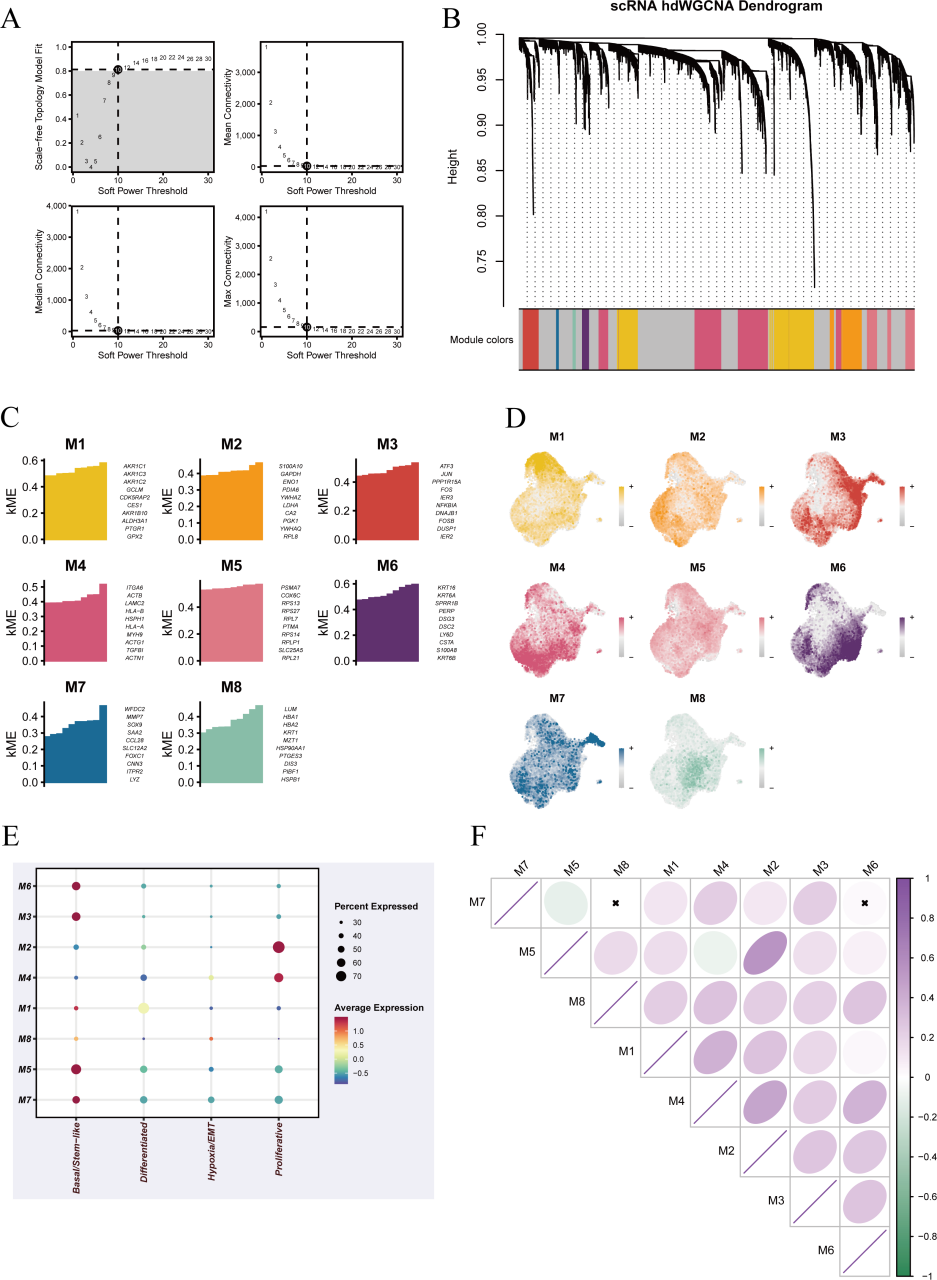
hdWGCNA of malignant epithelial cells. **(A)** Selection of the optimal soft-thresholding power. The scale-free topology fit index and mean connectivity were evaluated across a range of powers to determine the appropriate threshold for network construction. **(B)** Gene clustering dendrogram based on hdWGCNA. Modules containing genes with highly similar expression patterns are represented by different colors. **(C)** Heatmaps and module eigengene (ME) value distributions showing correlations between each module and four functional states (Hypoxia/EMT, Differentiated, Basal/Stem−like, and Proliferative), illustrating the expression trends of each module across states. **(D)** UMAP visualization of the distribution of module-specific genes, showing their spatial localization across malignant epithelial cells. **(E)** Bubble plot showing the average expression (color) and proportion of positive cells (size) for each module in the four functional states of malignant epithelial cells. **(F)** Module–module correlation matrix displaying Pearson correlation coefficients between modules, with * indicating significant correlations.

In this analysis, we first evaluated scale-free topology and mean connectivity under a range of soft-thresholding powers and selected power = 14 as the optimal parameter for network construction (**Figure 5A**). Genes were subsequently clustered hierarchically based on expression similarity, and dynamic tree cutting identified eight co-expression modules (M1–M8) (**Figure 5B**). Module eigengenes (hMEs) were then calculated to represent the principal expression patterns of each module, and their distribution across cell populations and samples was compared (**Figure 5C**). Mapping these module signals onto the UMAP space revealed their spatial localization and enrichment patterns within the single-cell atlas (**Figure 5D**). We next quantified module activity using hME/ModuleExprScore (UCell) and compared it among four functional states—Hypoxia/EMT, Differentiated, Basal/Stem-like, and Proliferative—via a bubble plot, where color indicates the mean module score and bubble size reflects the proportion of positive cells (**Figure 5E**). Notably, Basal/Stem-like cells exhibited strong associations with modules M3, M5, M6, and M7, whereas Proliferative cells were predominantly linked to modules M2 and M4. The other two states displayed generally low activity across most modules, suggesting functional specificity of these gene programs. Finally, the module–module correlation matrix revealed mostly low-to-moderate correlations between modules, with only a few module pairs showing strong associations, indicating relative independence of these co-expression programs (**Figure 5F**).

As a complementary analysis, we also visualized the co-expression networks and specific gene members of each module (**Supplementary Figure S2**). Hub genes with high network centrality may represent potential key regulators, and this visualization not only clarifies the actual gene composition of each module but also provides concrete candidates for subsequent functional validation and therapeutic target discovery.

### 3.6 Construction of the neoadjuvant therapy–associated malignant phenotype score

**Figure 6:**
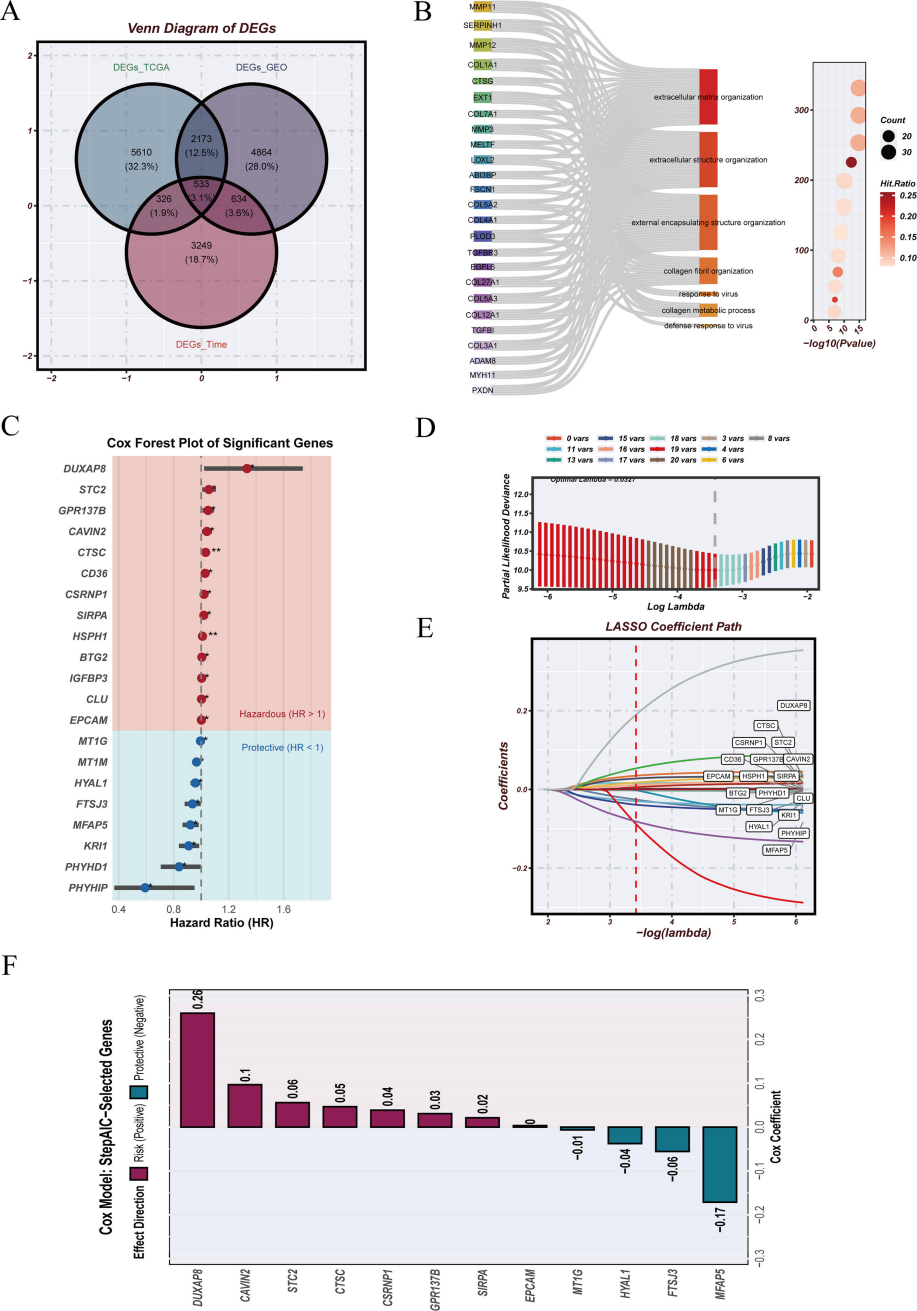
Construction of the neoadjuvant therapy–associated malignant phenotype score (NTAMPS). **(A)** Identification of the intersection genes shared between malignant epithelial cell markers and genes significantly associated with clinical response. **(B)** Functional enrichment analysis of the intersection genes, highlighting their involvement in key biological processes and pathways. **(C)** Results of univariate Cox regression on the intersection genes. **(D)** Feature selection of prognosis-related genes using the LASSO algorithm to construct NTAMPS. **(E)** Profiles of LASSO coefficients for the chosen genes under the optimal λ penalty, showing the shrinkage process. **(F)** Final NTAMPS gene coefficients, plotted with coefficient values on the X-axis and gene names on the Y-axis.

To construct the Neoadjuvant Therapy–Associated Malignant Phenotype Score (NTAMPS), differentially expressed genes (DEGs) were initially screened between pre- and post-treatment malignant epithelial cells using the FindAllMarkers function. These DEGs were then intersected with those from the TCGA-ESCA and validation cohort GEO datasets to obtain a set of candidate genes (**Figure 6A**). Subsequent enrichment analyses of the overlapping gene set were performed for Gene Ontology (GO) categories and Kyoto Encyclopedia of Genes and Genomes (KEGG) pathways (**Figure 6B**), revealing significant enrichment in tumor-related processes, covering processes such as extracellular matrix remodeling, cell–cell adhesion, epithelial–mesenchymal transition, and modulation of immune responses.

Univariate Cox regression was conducted on the candidate genes (**Figure 6C**), identifying 21 genes significantly associated with survival. These were further refined via least absolute shrinkage and selection operator (LASSO) regression(**Figures 6D, E**), with tenfold cross-validation used to determine the optimal penalty parameter (λ_1se = 0.09092128). The coefficient path plot demonstrated that most coefficients shrank toward zero as λ increased, leaving a stable subset of features. Stepwise regression following LASSO ultimately retained 12 genes, and their multivariate coefficients are shown in **Figure 6F**. NTAMPS was derived by summing the expression levels of these genes, each multiplied by its corresponding coefficient:

NTAMPS = 0.030709335×Expr(GPR137B) − 0.055510952×Expr(FTSJ3) + 0.046448669×Expr(CTSC) + 0.055827053×Expr(STC2) − 0.037636353×Expr(HYAL1) + 0.003378600×Expr(EPCAM) − 0.006280315×Expr(MT1G) + 0.038432891×Expr(CSRNP1) + 0.096635546×Expr(CAVIN2) − 0.171176051×Expr(MFAP5) + 0.021303487×Expr(SIRPA) + 0.259693553×Expr(DUXAP8).

Overall, this process integrated treatment-associated transcriptomic alterations, prognostic relevance, and regularized modeling at the malignant epithelial cell level, resulting in a quantifiable NTAMPS that provides a robust basis for subsequent risk stratification and prognostic assessment.

### 3.7 Prognostic performance and risk stratification of the NTAMPS in the training and independent validation datasets

**Figure 7:**
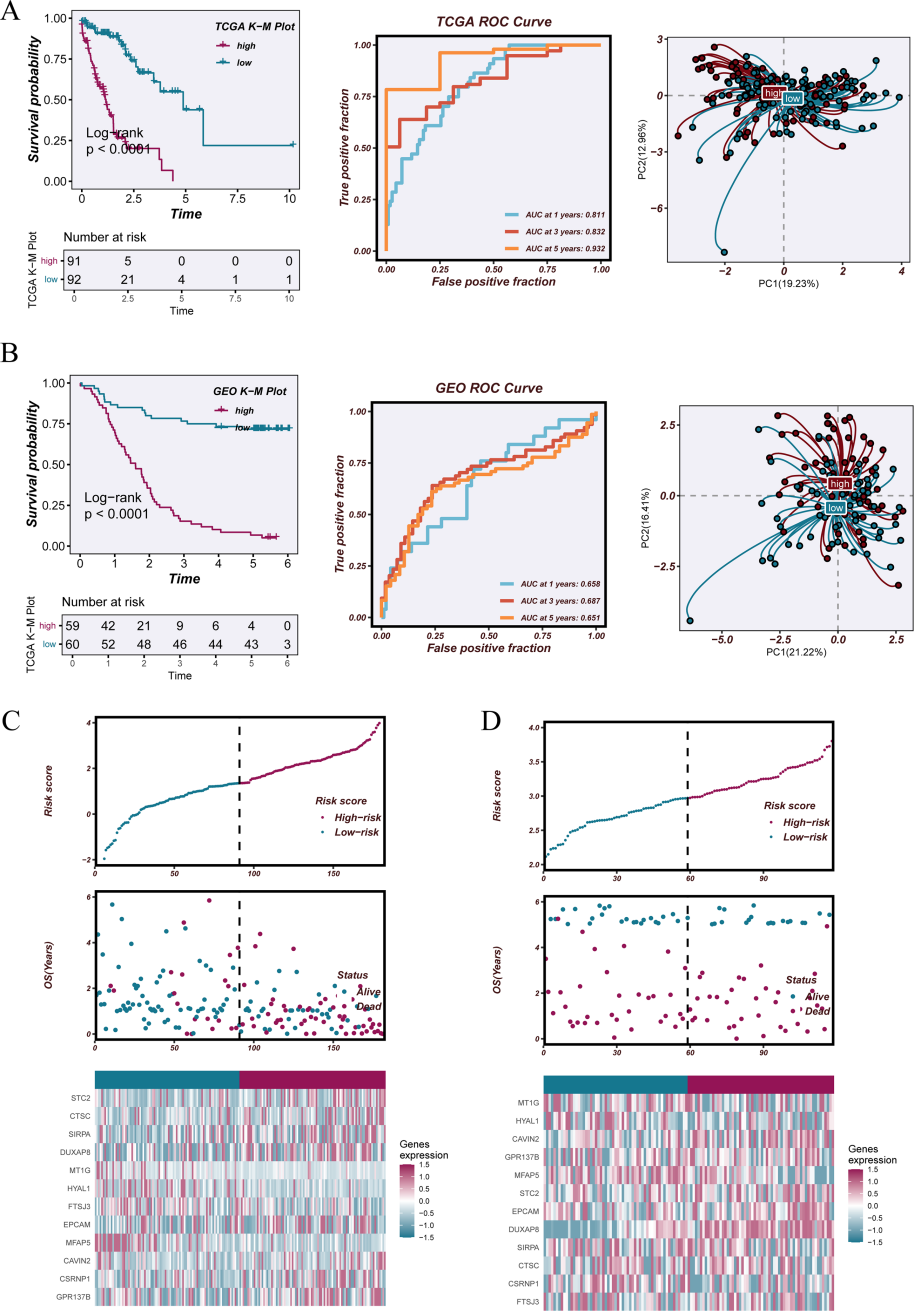
Prognostic performance and risk stratification of the NTAMPS in the training and validation cohorts. **(A)** Kaplan–Meier survival analysis for overall survival between high- and low-risk groups in the TCGA-ESCA cohort. Time-dependent ROC curves for 1-, 3-, and 5-year survival prediction, and principal component analysis (PCA) plots showing the distribution of patients in high- and low-risk groups based on the NTAMPS. **(B)** Kaplan–Meier survival plots contrasting high- and low-risk groups in the GSE53624 validation set. Time-dependent ROC curves for 1-, 3-, and 5-year survival, together with PCA visualizations highlighting the distinction between the two risk categories. **(C)** Visualization of risk score distribution, survival outcome scatter plots, and expression heatmap of the 12 NTAMPS genes within the TCGA-ESCA cohort. **(D)** Graphs depicting risk score distribution, survival status plots, and the expression heatmap of the 12 NTAMPS genes in the GSE53624 cohort.

Within the TCGA-ESCA training cohort, patients were divided into high- and low-risk categories based on the median NTAMPS score. Kaplan–Meier analysis revealed significantly poorer overall survival in the high-risk group relative to the low-risk group (P < 0.001) (**Figure 7A**, left). The time-dependent ROC analysis produced AUC values of 0.811, 0.832, and 0.932 for predicting 1-, 3-, and 5-year survival, respectively, indicating strong prognostic performance (**Figure 7A**, middle). Principal component analysis (PCA) displayed distinct clustering of the two risk subgroups in the two-dimensional space, highlighting the strong discriminative power of NTAMPS (**Figure 7A**, right). For the GSE53624 validation cohort, patients were likewise grouped into high- and low-risk categories according to the median risk score. Consistent with the training set results, the high-risk group showed markedly reduced survival relative to the low-risk group (P < 0.0001) (**Figure 7B**, left). The time-dependent ROC analysis yielded AUC values of 0.658, 0.687, and 0.651 for predicting 1-, 3-, and 5-year survival, respectively (**Figure 7B**, middle). Principal component analysis (PCA) further demonstrated distinct clustering of patients between the two risk groups (**Figure 7B**, right). Plots of risk score distribution and survival status further illustrated that mortality proportion rose with increasing risk scores (**Figures 7C, D**, top and middle panels). The heatmaps displayed increased expression of several risk-associated genes and reduced expression of protective genes within the high-risk group, further confirming the molecular distinctions captured by NTAMPS (**Figures 7C, D**, bottom panels).

### 3.8 Prognostic performance and risk stratification for NTAMPS across training and validation datasets

**Figure 8:**
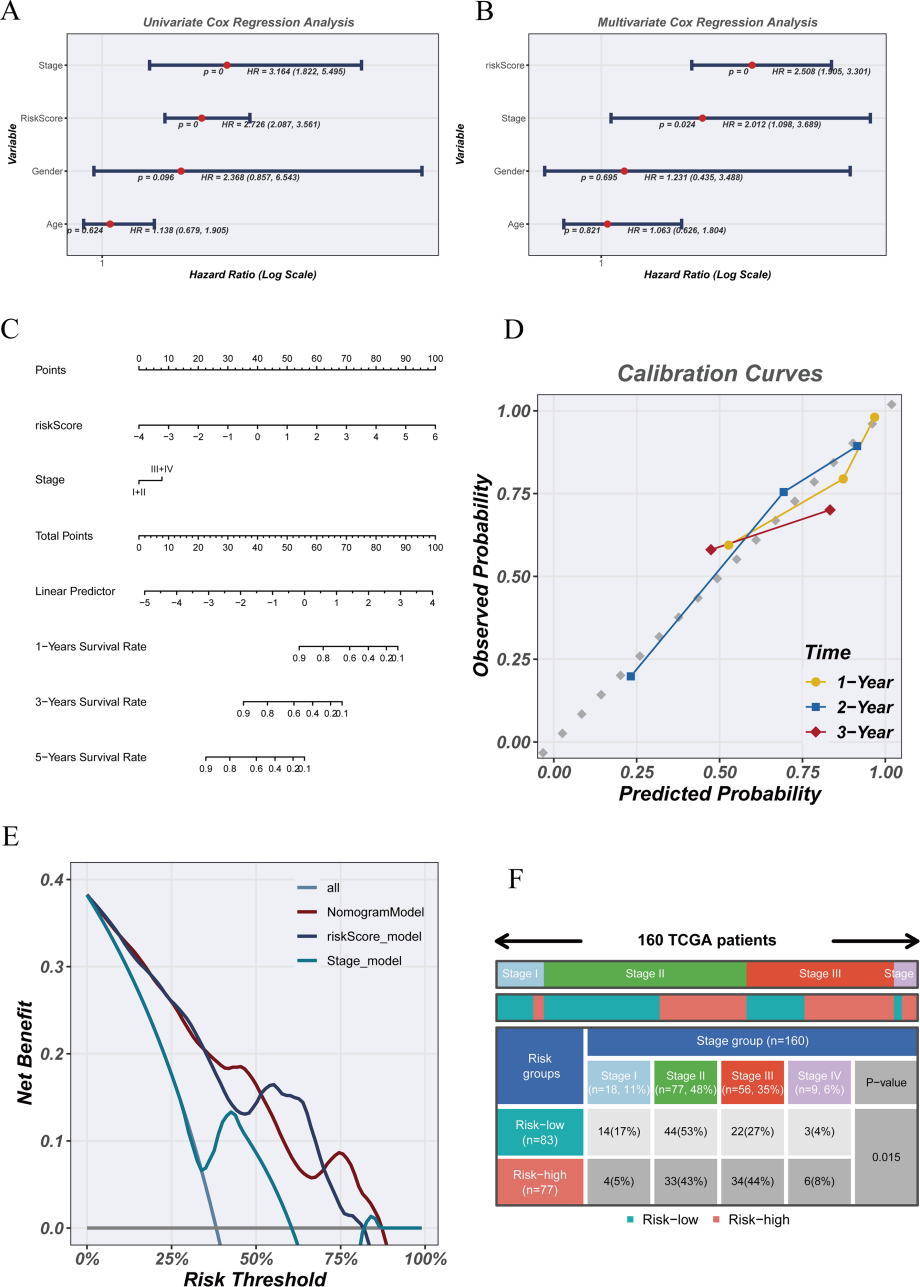
Integration of NTAMPS and clinical features for nomogram-based prognostic assessment. **(A)** Univariate Cox regression analysis assessing the association between the NTAMPS risk score and clinical features with overall survival. **(B)** Multivariate Cox regression analysis validating the robustness of NTAMPS risk score as an independent prognostic factor. **(C)** Nomogram constructed based on the NTAMPS risk score and clinical stage to predict 1-, 3-, and 5-year survival probabilities. **(D)** Calibration plots illustrating the concordance between model-predicted survival probabilities and observed outcomes. **(E)** Decision curve analysis (DCA) evaluating the net clinical benefit of the nomogram, risk score, and clinical stage models over a range of risk thresholds. **(F)** Proportion of patients in high- and low-risk groups stratified by clinical stage.

In the TCGA-ESCA cohort, univariate Cox regression analysis (**Figure 8A**) revealed that both the NTAMPS risk score (HR = 2.726, 95% CI: 2.087–3.561, p < 0.001) and clinical stage (HR = 3.164, 95% CI: 1.822–5.495, p < 0.001) were significantly associated with overall survival. Multivariate Cox regression analysis (**Figure 8B**) also verified that the NTAMPS risk score (HR = 2.508, 95% CI: 1.905–3.301, p < 0.001) together with clinical stage (HR = 2.012, 95% CI: 1.098–3.689, p = 0.024) served as independent prognostic indicators. Using these two variables, we constructed a nomogram (**Figure 8C**) to estimate survival probabilities at 1, 3, and 5 years. The calibration curves (**Figure 8D**) showed strong concordance between predicted and actual survival outcomes, indicating favorable predictive accuracy. Decision curve analysis (DCA) (**Figure 8E**) further indicated that combining NTAMPS with clinical stage yielded notable net clinical benefit across multiple threshold probability ranges. The distribution of patients classified as high- or low-risk across clinical stages is illustrated in **Figure 8F**, revealing a significantly greater proportion of high-risk cases in Stage III–IV relative to low-risk cases (p < 0.001).

### 3.9 Evaluation of immunotherapy response and anticancer drug susceptibility

To further assess the possible clinical applicability of NTAMPS for guiding treatment decisions, we carried out assessments of predicted immunotherapy responsiveness and drug sensitivity. Immunotherapy efficacy prediction helps identify the likelihood of response among patients with different risk levels, thereby optimizing patient stratification and therapeutic evaluation. Drug sensitivity analysis reveals differences in predicted responses to various targeted and chemotherapeutic agents, providing guidance for personalized treatment.

**Figure 9:**
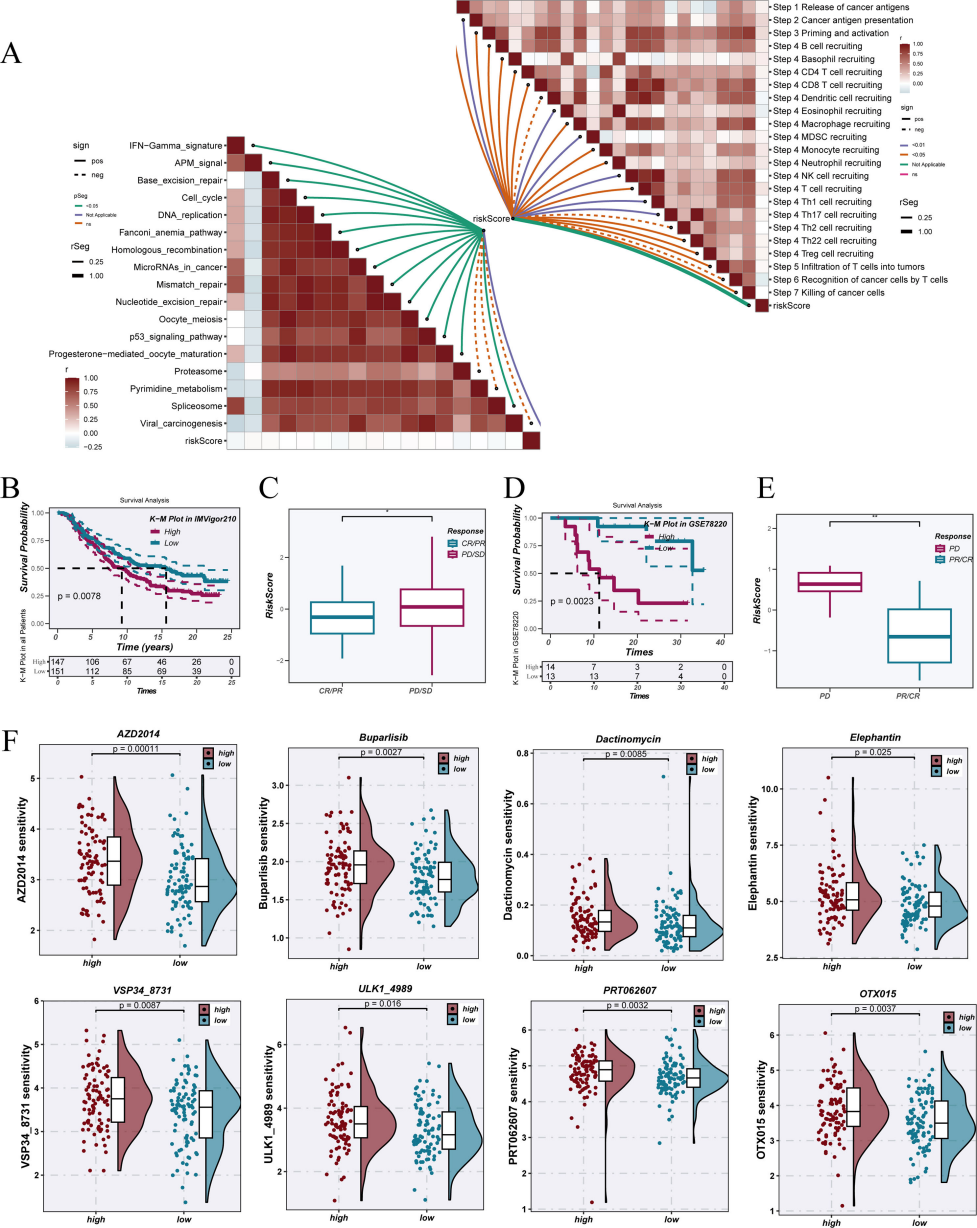
Evaluation of predicted immunotherapy response and analysis of anticancer drug sensitivity. **(A)** Butterfly plot showing correlations between NTAMPS and immune_cycle as well as immunotherapy predicted pathways, calculated using the ssGSEA algorithm. **(B)** Kaplan–Meier survival plots comparing high- versus low-NTAMPS groups within the IMvigor210 cohort. **(C)** Differences in NTAMPS between immune therapy response subgroups in the IMvigor210 cohort. **(D)** Survival curves for high- and low-NTAMPS patients in the GSE78220 cohort. **(E)** Differences in NTAMPS between immune therapy response subgroups in the GSE78220 cohort. **(F)** Drug sensitivity analysis results showing differences in predicted drug sensitivity between high- and low-NTAMPS patients.

Correlation analysis based on the single-sample GSEA (ssGSEA) method (**Figure 9A**) revealed a strong association between NTAMPS and multiple immune cycle processes and immunotherapy-predicted pathways, including key steps such as antigen release, antigen presentation, T-cell recruitment, and expression of immunosuppressive molecules. In the IMvigor210 immunotherapy cohort, high NTAMPS patients had notably shorter overall survival than those with low NTAMPS (**Figure 9B**). Moreover, NTAMPS scores were markedly elevated in non-responders compared with responders (**Figure 9C**). In the GSE78220 immunotherapy cohort, low NTAMPS patients demonstrated more favorable survival outcomes (**Figure 9D**), and the NTAMPS scores were substantially greater among non-responders than responders (**Figure 9E**).

Drug sensitivity analysis (**Figure 9F**) indicated clear variation of predicted sensitivity when comparing high-NTAMPS to low-NTAMPS patients for several drugs, including AZD2014, Buparlisib, Dactinomycin, and Elephantin, suggesting that NTAMPS may serve as an important stratification indicator for guiding personalized therapy.

### 3.10 DUXAP8 as the top-ranking risk gene: expression profiling and functional characterization in ESCC

**Figure 10:**
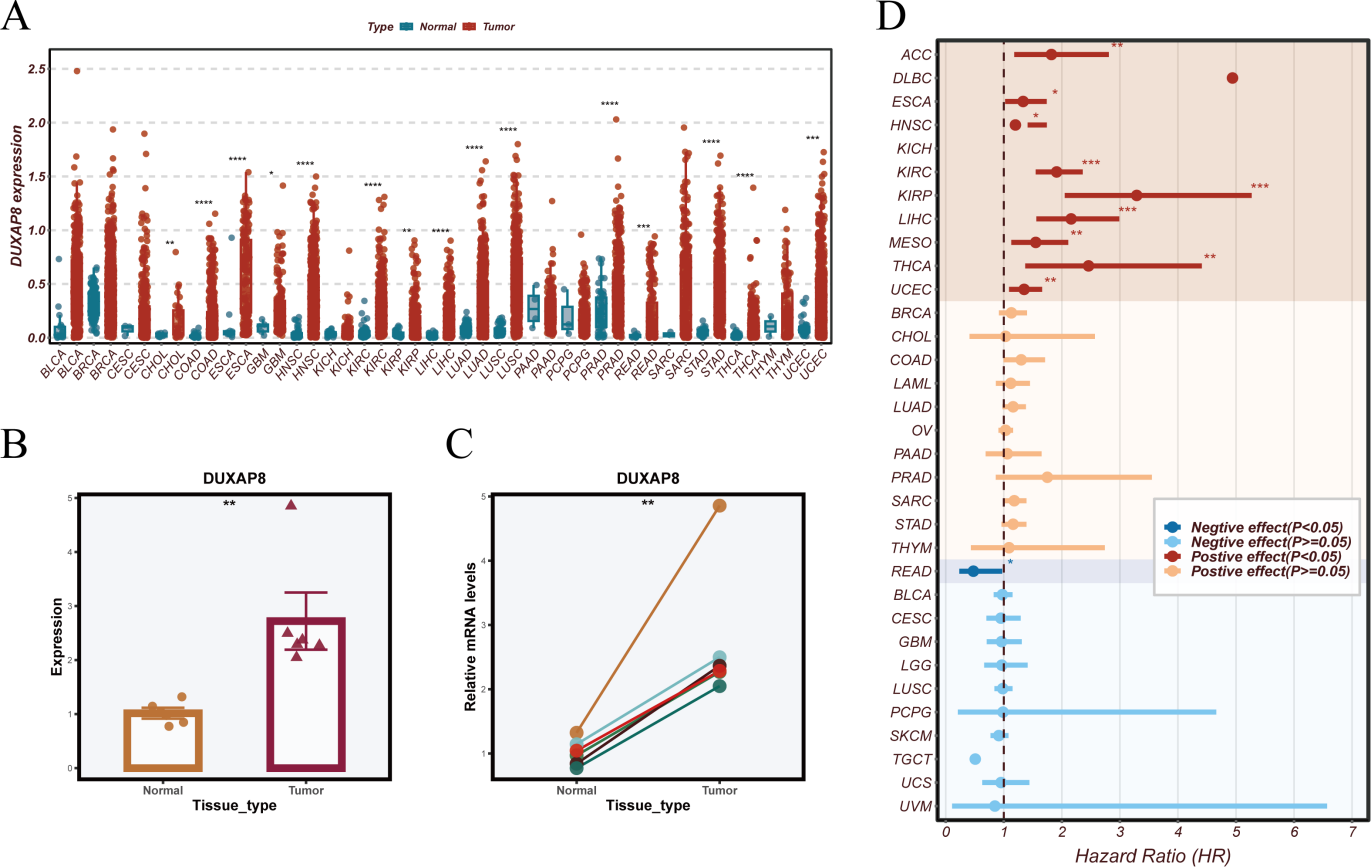
Validation of DUXAP8 expression and prognostic analysis. **(A)** Pan-cancer analysis using TCGA datasets revealed variations in DUXAP8 expression between malignant and adjacent normal tissues across diverse cancer types. **(B)** RT-PCR was used to measure DUXAP8 expression in paired tumor and adjacent normal tissues from ESCC specimens obtained at our institution. **(C)** Paired PCR results comparing DUXAP8 expression in matched ESCC tumor and adjacent normal tissues. **(D)** Forest plot illustrating the prognostic impact of DUXAP8 across different cancer types, with hazard ratios (HR) used to assess its effect on patient survival.

In the prognostic model constructed in this study, DUXAP8 exhibited the highest risk coefficient (0.26), suggesting a potentially critical role in disease progression; therefore, it was selected for further investigation. Pan-cancer analysis revealed that DUXAP8 was significantly upregulated in multiple tumor types, including esophageal squamous cell carcinoma (ESCC) (**Figure 10A**). RT-PCR validation in ESCC tissue samples collected from our cohort demonstrated markedly elevated DUXAP8 expression in tumor tissues relative to paired adjacent normal counterparts (**Figure 10B**), a finding further confirmed by paired PCR analysis (**Figure 10C**). Prognostic analysis indicated that DUXAP8 levels showed a significant correlation with patient survival across multiple cancer types (**Figure 10D**).

**Figure 11:**
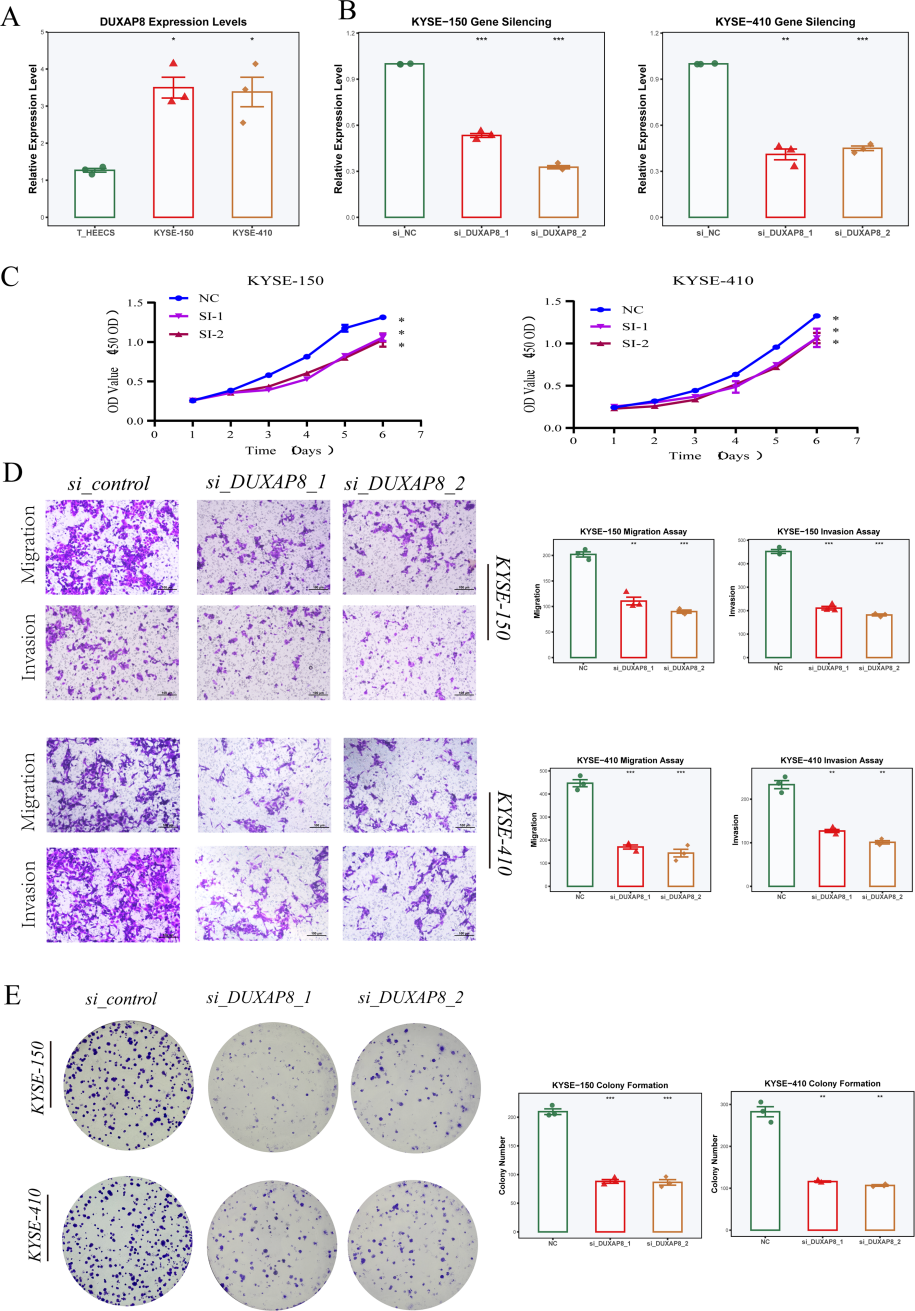
Expression and functional validation of DUXAP8 in ESCC cells. **(A)** RT-PCR analysis of DUXAP8 expression in the normal esophageal epithelial cell line (T_HEECS) and two esophageal squamous cell carcinoma (ESCC) cell lines (KYSE-150 and KYSE-410). **(B)** Verification of siRNA-mediated DUXAP8 knockdown efficiency in KYSE-150 and KYSE-410 cells following transfection with si_DUXAP8_1 and si_DUXAP8_2. **(C)** CCK-8 assays were performed to assess how silencing DUXAP8 influences proliferation in KYSE-150 and KYSE-410 cell lines. **(D)** Transwell migration and invasion experiments examined changes in motility and invasiveness following DUXAP8 depletion in these cells. **(E)** Colony formation assays measured alterations in clonogenic potential after DUXAP8 knockdown in KYSE-150 and KYSE-410 cells.

To clarify the functional role of DUXAP8 in ESCC, we assessed its expression in a normal esophageal epithelial cell line (T_HEECS) and two ESCC lines (KYSE-150 and KYSE-410), finding notable upregulation in tumor-derived cells (**Figure 11A**). Two independent siRNAs were used to silence DUXAP8 in both KYSE-150 and KYSE-410, with qRT-PCR confirming efficient knockdown (**Figure 11B**). CCK-8 assays indicated that DUXAP8 knockdown markedly reduced cell proliferation in both lines (**Figure 11C**). Transwell assays revealed that silencing DUXAP8 substantially decreased migration and invasion capacities (**Figure 11D**). Furthermore, colony formation assays showed that knockdown of DUXAP8 significantly diminished the clonogenic potential of ESCC cells (**Figure 11E**). Collectively, our results suggest that DUXAP8 is abundantly expressed in ESCC and facilitates proliferation, migration, invasion, and colony-forming ability, suggesting a potential oncogenic function in ESCC development.

## 4 Discussion

ESCC represents a significant component of the worldwide cancer burden, showing especially high prevalence in East Asia and certain African regions (30). The majority of cases are detected at advanced stages, which is associated with unfavorable prognoses. Although neoadjuvant chemotherapy, chemoradiotherapy, and immunotherapy have been integrated into clinical practice, overall efficacy is still limited and interpatient variability remains substantial (31). Recent phase III neoadjuvant trials (**Supplementary Table 3**) illustrate substantial variability in pathological response across different regimens, with nCRT generally achieving the highest pCR rates and nICT also providing clinically meaningful tumor regression. In parallel, several studies have reported that nICT is associated with a more favorable and manageable toxicity profile compared with conventional nCRT, making it an increasingly attractive neoadjuvant option for resectable ESCC (32–35). However, response to nICT remains highly heterogeneous, and no validated biomarkers currently exist to identify patients who are most likely to benefit. This critical gap underscores the need for single-cell–level dissection of malignant and immune cell states, which provides unique advantages for uncovering treatment-relevant phenotypes and enabling the development of precise predictive markers. Accurately identifying high-risk patients and elucidating the biological mechanisms underlying treatment response are pressing clinical challenges. The heterogeneity of ESCC extends beyond histopathology to dynamic changes in the tumor microenvironment (TME) and intercellular communication networks (36). scRNA-seq enables high-resolution characterization of diverse cell populations and functional states, offering unique advantages in dissecting TME remodeling and uncovering molecular drivers with prognostic and therapeutic potential (37).

This study developed a single-cell landscape of ESCC encompassing samples from both pre- and post-neoadjuvant therapy phases, capturing TME remodeling and transcriptional reprogramming of malignant epithelial cells under therapeutic pressure. Leveraging these data, NTAMPS was developed to stratify prognosis and investigate therapy-associated biological links. Importantly, analysis revealed a marked reorganization of the MIF signaling network and functional alterations in malignant epithelial cells, with DUXAP8 identified as the top-weighted gene within NTAMPS and validated for its oncogenic role.

The MIF signaling axis is a highly conserved immune regulatory pathway originally identified for its ability to inhibit macrophage migration (38). MIF is produced by immune cells, epithelial cells, and stromal fibroblasts, and binds to receptors such as CD74—often complexed with CD44—or chemokine receptors including CXCR2, CXCR4, and ACKR3 (39). These interactions trigger MAPK–ERK and PI3K–AKT signaling cascades, influencing cell survival, motility, inflammatory responses, and immunosuppression (40, 41). In multiple cancer types, MIF contributes to tumor initiation, angiogenesis, immune evasion, and therapeutic resistance, partly through remodeling of the TME (42). Elevated MIF expression correlates with adverse prognosis, highlighting its potential utility as both a diagnostic and prognostic biomarker, as well as a candidate therapeutic target.

Our CellChat-based comparative analysis revealed that among the communication pathways most affected by therapy—including SPP1, PARs, ESAM, MIF, and MHC-I/II—the MIF-associated ligand–receptor pairs MIF–ACKR3, MIF–(CD74+CXCR4), and MIF–(CD74+CD44) showed enhanced or more intricate interactions after treatment. Fibroblasts consistently acted as dominant “signal senders,” while macrophages were the primary “signal receivers” both before and after therapy. These findings align with prior evidence implicating the MIF/CD74/CD44 and MIF/CXCR4 axes in immunosuppression, macrophage reprogramming, and tumor-promoting stroma formation. While direct experimental confirmation of this pathway in ESCC remains limited, our findings indicate that MIF could participate in the remodeling of immune and stromal components following neoadjuvant therapy, warranting further investigation. Compared with earlier ESCC single-cell studies, our dataset incorporates several design and data-quality advantages. Previous work has typically profiled a limited number of tumor samples without neoadjuvant treatment exposure and often inferred immunologic features from baseline tumor states alone (43). Other studies have included normal–tumor paired samples but lacked pre- and post-treatment comparison, focusing mainly on predicting immunotherapy response from specific immune subsets such as dendritic cells (17). In our study, we applied stringent quality-control thresholds (500 ≤ nFeature_RNA ≤ 10,000) and, importantly, incorporated paired pre- and post-NAT samples, enabling direct assessment of therapy-induced shifts in malignant phenotypes, immune infiltration, and intercellular signaling. This design captures clinically relevant dynamic remodeling and provides the resolution necessary to reveal treatment-driven rewiring of the MIF axis, a phenomenon not detectable in prior datasets.

Malignant epithelial cell analysis with inferCNV identified a subset harboring large-scale copy number variations, which were classified into four functional states: Hypoxia/EMT, Differentiated, Basal/Stem-like, and Proliferative. Post-treatment, the Basal/Stem-like fraction expanded while the other three states declined, indicating a potential survival advantage for dedifferentiated phenotypes under therapeutic stress. Pseudotime and transcription factor network analyses supported these transitions, revealing adhesion/junction assembly programs enriched in late stages and metabolic/xenobiotic response programs in early stages. Functional module identification highlighted Basal/Stem-like (M3/M5/M6/M7) and Proliferative (M2/M4) states as key drivers of therapy adaptation.

By integrating pre–post treatment differentially expressed genes with survival associations, we developed NTAMPS, demonstrating consistent prognostic accuracy in both the TCGA-ESCA training set and the GSE53624 validation set. In immunotherapy datasets, high NTAMPS was associated with worse outcomes and was enriched in non-responders, while also correlating with multiple immune cycle and immunotherapy-predictive pathways. Analysis of drug response profiles suggested distinct sensitivities to targeted drugs when comparing high- versus low-risk groups, offering potential guidance for personalized treatment.

DUXAP8, a long non-coding RNA, had the highest positive risk coefficient in NTAMPS. Previous studies have shown that DUXAP8 promotes proliferation, migration, invasion, and EMT in various cancers, with overexpression linked to poor prognosis (44–47). In ESCC, it has been reported to be upregulated and to enhance tumor aggressiveness. Our pan-cancer and in-house analyses confirmed DUXAP8 overexpression, and siRNA-mediated knockdown in ESCC cell lines suppressed proliferation, migration, invasion, and colony formation, supporting its role as a functional driver within the NTAMPS framework. Although many prognostic frameworks rely on multi-gene or gene-pair–based ranking strategies to enhance robustness across heterogeneous datasets, particularly through relative expression comparison rather than absolute abundance, as demonstrated in prior studies (48), DUXAP8 consistently emerged as the dominant survival-associated transcript across malignant states, trajectories, and bulk cohorts in our analysis. This convergence provides a biological rationale for adopting a streamlined single-gene–anchored model within NTAMPS, offering complementary advantages in interpretability and clinical applicability rather than functioning as an alternative to gene-pair classifiers.

Limitations of this study include reliance on public datasets and a relatively small in-house validation cohort. The mechanistic role of the MIF axis in post-treatment immune and stromal remodeling, as well as the upstream regulation and downstream effectors of DUXAP8, require further experimental elucidation. Although our functional assays support the biological contribution of DUXAP8 to malignant behaviors, establishing whether its association with survival reflects a causal effect or a correlated molecular phenotype will require more rigorous causal-inference frameworks (49). Recent methodological advances—including Mendelian-randomization–based analyses and genetically anchored perturbation models—provide promising strategies to dissect the directionality of gene–phenotype relationships and may be applied to future evaluations of NTAMPS components (50). Future research integrating spatial transcriptomics, multi-omics profiling, and functional perturbations may refine our understanding of NTAMPS components and facilitate its clinical translation for prognosis and therapeutic decision-making in ESCC.

## Data Availability

The original contributions presented in the study are included in the article/**Supplementary Material**. Further inquiries can be directed to the corresponding author.

## Supplementary material

Supplementary Figure 1 Overview of quality control indicators and marker gene expression profiles. **(A)** Gene number (nFeature_RNA), transcript abundance (nCount_RNA), and mitochondrial content (pMT) distribution across all samples prior to filtering. **(B)** Corresponding distributions after applying quality control filters. **(C)** Cluster-wise expression patterns of selected marker genes, where dot size reflects the proportion of expressing cells and color depth denotes mean expression intensity.

Supplementary Figure 2 Visualization of gene co-expression networks for each hdWGCNA module. The figure displays the intramodular connectivity patterns of genes in the eight identified hdWGCNA modules (M1–M8), with each node representing a gene and edges indicating significant co-expression relationships. Nodes are colored according to their assigned module, and edge density reflects the strength of co-expression. Highly connected hub genes are positioned centrally within each module network.
