# Supplementary figures and images for: Neoadjuvant therapy–associated malignant phenotype score predicts prognosis and highlights the roles of MIF signaling and DUXAP8 in ESCC
Source: Front Immunol. 2026 Jan 14;16:1683349. doi: 10.3389/fimmu.2025.1683349 (PMC12847432; doi:10.3389/fimmu.2025.1683349)

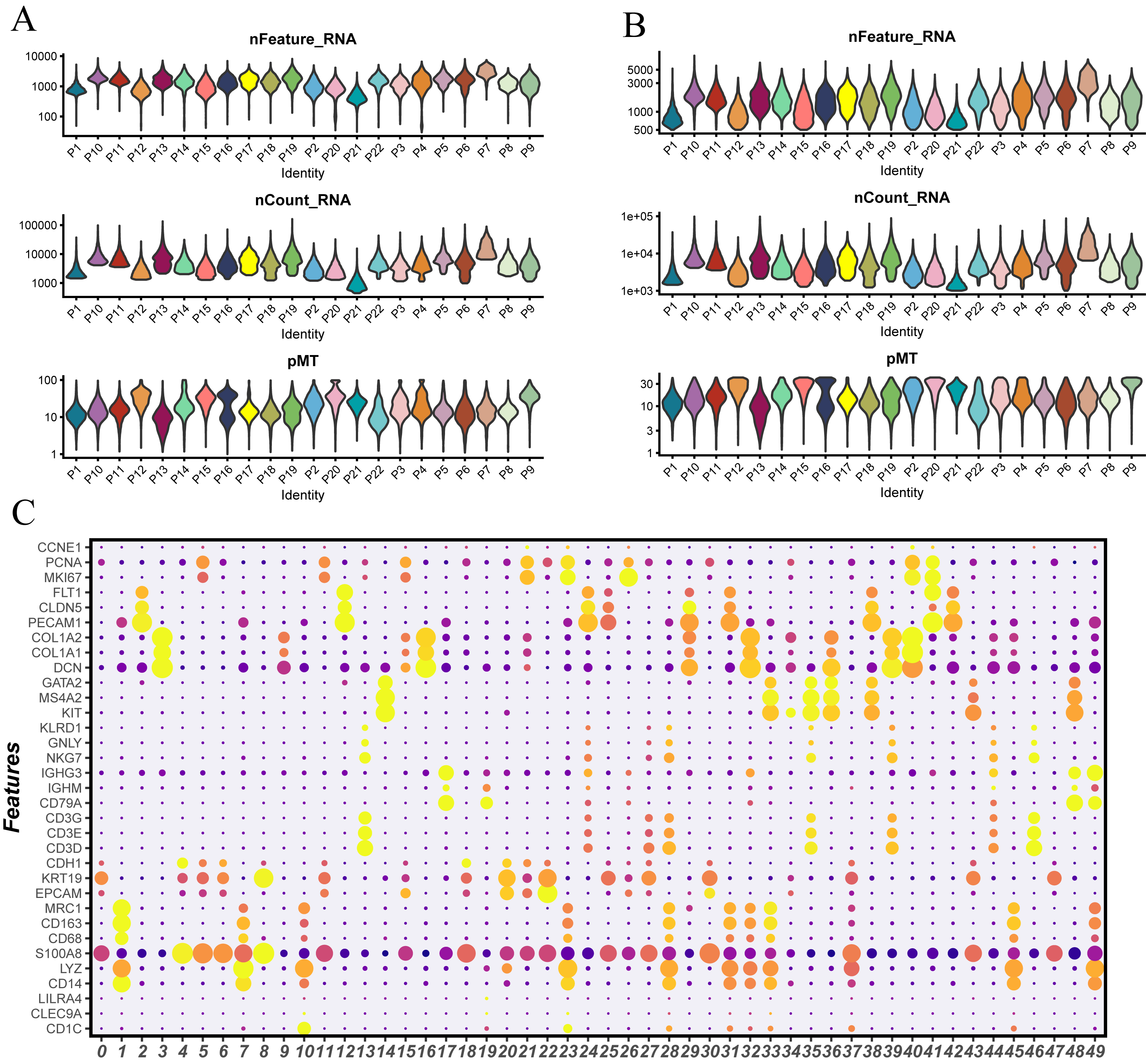

Supplement: Supplementary Figure 1 — Overview of quality control indicators and marker gene expression profiles. (A) Gene number (nFeature_RNA), transcript abundance (nCount_RNA), and mitochondrial content (pMT) distribution across all samples prior to filtering. (B) Corresponding distributions after applying quality control filters. (C) Cluster-wise expression patterns of selected marker genes, where dot size reflects the proportion of expressing cells and color depth denotes mean expression intensity. [file Image1.tif]

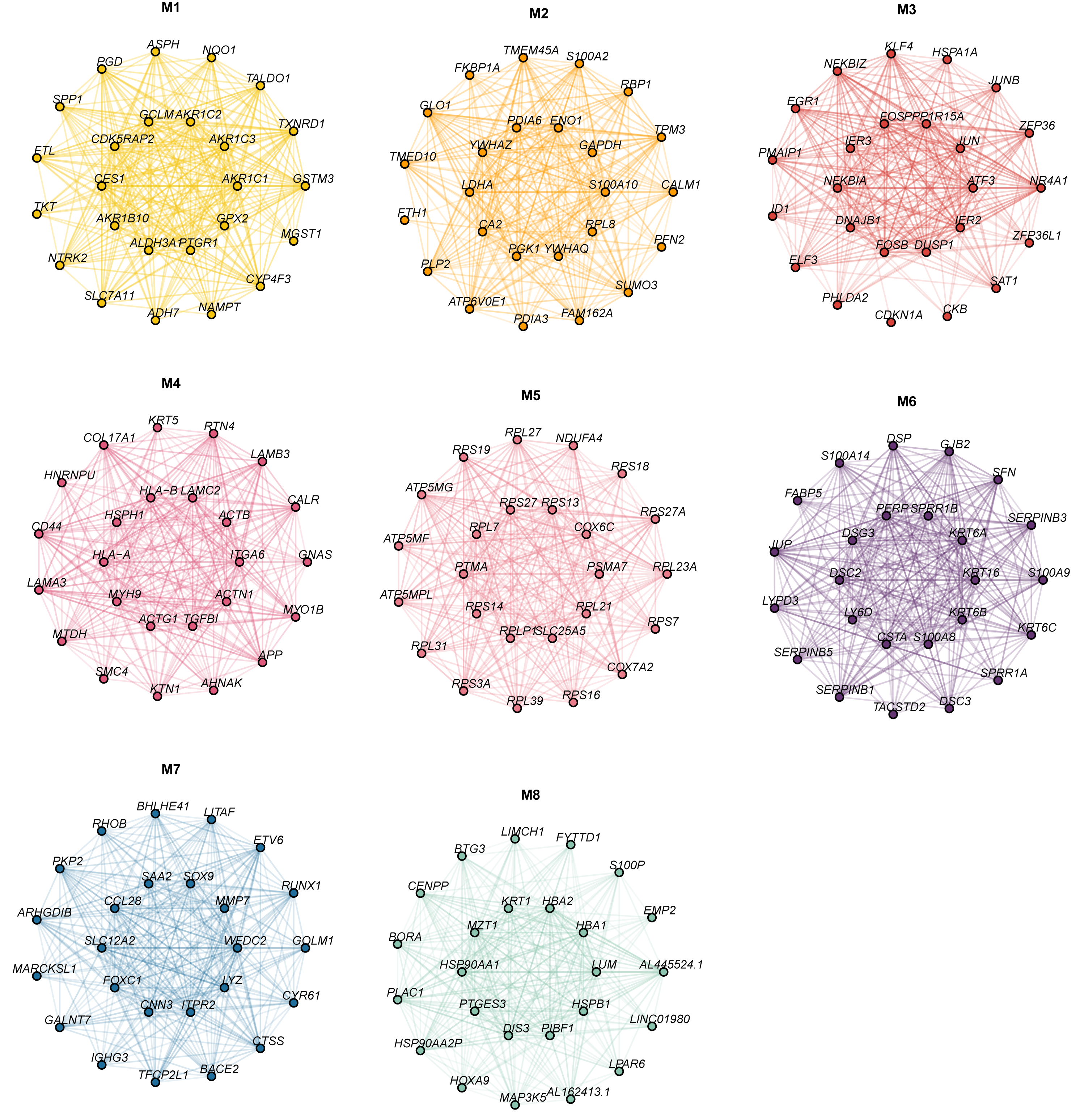

Supplement: Supplementary Figure 2 — Visualization of gene co-expression networks for each hdWGCNA module. The figure displays the intramodular connectivity patterns of genes in the eight identified hdWGCNA modules (M1–M8), with each node representing a gene and edges indicating significant co-expression relationships. Nodes are colored according to their assigned module, and edge density reflects the strength of co-expression. Highly connected hub genes are positioned centrally within each module network. [file Image2.tif]
